# Oxygen and Protoplasm

**Published:** 1882-04

**Authors:** W. H. Pitt


### Oxygen and Protoplasm.

By W. H. Pitt, M. D.

The discovery of oxygen a little over a hundred years ago by Dr. Priestley, of England, marked a new era in the history of science. But notwithstanding the vast addition to human knowledge in reference to the natural world which his great discovery afforded to all, Priestley, like many a naturalist before him, suffered persecution. A century passed, and Dr. Draper, of this country, discovered oxygen in the sun. The land of Priestley was the first to do him honor, while all the world applauded. But in considering that one discoverer had oxygen all around him, and in almost every compound within reach, we must bear in mind that the other explorer found it 91,000,000 miles away.

The difference in the length of the human arm as a radius compared with the radius vector of the sun, is truly wonderful, but no more surprising than the comparison between ancient and modern thought as applied to natural phenomena.

It is now generally understood that the "universe is governed by law," and not subject to the caprice of chance or impossibilities, built up in the brain of dreamers. We have come to a point now, thanks to scientific courage, when facts are fearlessly declared, and when, without fear or hindrance, all in common may attempt to find out the laws which govern them.

These laws, however, particularly in chemico-physics, are so intricate, because dealing for the most part with the immeasurably small molecules and atoms, that our limited senses fail and we are left to reason from analogy or in utter darkness.

The theory that all matter may be reduced to ultimate elementary atoms, goes a great way towards explaining the mysterious processes constantly at work in organic nature. The principal inorganic molecules $H_2O$ water, $CO_2$ carbonic acid, $H_3N$ ammonia, every body will admit are formed from the elementary substances, oxygen, hydrogen, carbon, nitrogen. The complex organic molecule contains them all, and in decomposing is resolved into these simpler inorganic compounds.

The consistent reasoner is forced to the conclusion that the complex is made up of the simple. It is also evident that before the complex were fully formed, the simple molecules were, and that before these existed, matter was in an elementary state. Whether, however, the first organic molecule was formed by the union of these simpler ones, and thus passing the boundary of dead matter, sprang into being, forming a nucleus around which many millions might cluster, and thus differentiated into organisms, or whether it was brought around some other way, is not positively known.

That such a view of chemical organization into being does not detract from the supposed plan or design manifested in a First Cause in which so many have been taught to believe, but rather ennobles it, increasing our knowledge of the mystery of life, is an idea prevailing largely at the present time.

But the object of this paper is to direct more earnest attention to oxygen, to ask you to note the part it plays in making our earth what it is, teeming with animal and vegetable life.

The elements of which the earth is composed were originally in a free state, and through chemical union and contraction brought around the present appearance of things. In this combination of elements to form other matter of entirely different properties from its constituents, oxygen took a more prominent part than any of the other elements. Thus we find one half the weight of the solid earth, eight-ninths the weight of water, and one-fifth of the atmosphere, to be oxygen gas either in a combined or free state. The great affinity which it manifests for the other elements singles it out as the one particular element with which nature forms the greatest number and variety of co[illegible]pounds. It does not seem so surprising, then, that since we find it the chief earth-making element, that we should also discover it to be the life-giving and the life-sustaining element.

The infinite phenomena observed in matter, whether in an elementary or a compound state, are the struggles of nature to reach an equilibrium. This equilibrium is never attained either in the atom or the mass, hence the restless state of matter, living or dead, which we nominate motion, force, life. It is this unbalanced state of oxygen which we call affinity, and which not only makes the earth possible, but all that lives thereon. In a world like ours it is wonderfully adapted to evolve light and heat, which are the constant disturbers of all static tendencies in matter.

And so ages before any life appeared the office of oxygen was to burn the elements, that out of the ashes might be formed our wondrous earth. Long after the conflagration, when the burnt metals and non-metals had become the acids, bases and salts, and the flaming hydrogen the oceans, the globe swung on with its black, dense atmosphere silently and in death.

The air becomes transparent; light breaks upon the scene; the golden rays smite the lifeless seas and the lifeless shores. Oxygen, like a conqueror, lingers in the air, and shall yet give vitality and beauty to the desolate new world.

At length the earth is clothed in verdure, flowers and fruit. The tepid waters swarm with life, and the clear atmosphere rests upon the sea. On the uplifted continents innumerable beings move bathed in the vital oxygen and the morning sun. Chemical and physical forces built up our planet; chemical and physical forces constitute and condition all its vitality.

The sequence is unbroken in which the potency of oxygen rendered possible the globular mass and then peopled its circumference with myriads of living beings. Without this element it is impossible to contemplate what the earth and its inhabitants could have been according to the present laws of nature.

That the living matter is made up from the dead, and that the living is constantly becoming dead, we have abundant evidence. The progress downwards of organic matter into inorganic is quite well understood—not so with the progress upwards.

The border line between the two distinctions we recognize in matter, may not be very wide; but never yet has a lover of nature been able to cross it and witness the evolution, or origin of life. Nor is this strange, since all knowledge, which is genuine, comes through sensation, and the senses of man are limited.

As far as our senses do reach, however, it is safe to go and affirm whatever may be true in examining either the highest or the lowest organisms.

The bodies of very low animal types contain a fluid, structureless and jelly-like, called *sarcode.* A similar substance is also found in plants and is called *protoplasm.* The sarcode of the animal and the protoplasm of the plant have been proved to be identical, and are believed by many naturalists to be "the physical basis of life." Certain it is that whatever lives, contains protoplasm. "Where there is life, there is protoplasm."

But, strange to say, it may exist outside of the body of the plant or animal, and so has a kind of independent life all by itself. It is a glairy liquid, resembling the white of the egg. Chemical analysis shows it to be an albuminous compound, composed of oxygen, hydrogen, carbon, nitrogen.

You place a point of it under the microscope and note its movements, as it is never still. From its surface may be thrust out finger-like projections, called pseudopodia. Again, portions of it will flow off in thread-like streams, run together and finally be contracted into the central mass. At other times undulations pass over it, and quickly its whole form is changed, apparently by some internal force. It is certainly living m tter, but an organism without organs, as it has no mouth or digestive apparatus. It throws out a stream of itself to capture and assimilate food. These wonderful beings have been generalized under the name *monera,* because of their apparent homogenous structure and singleness of form.

There are different varieties, some with a well defined nucleus in the center, and these are called *amœba.*

Haeckel found in the fresh water of Jenna small lumps of living protoplasm, and probably similar organisms may be found in the fresh water at Buffalo. But this living slime, which seems to be linked so near the inorganic world, will die in a vacuum and become as motionless as the white of the egg which it so closely resembles.

And just here I am forced to the conclusion that oxygen is the organizer of its molecules and its stimulating energy. With the ever free and ever ready oxygen as a nucleus, its albuminoid molecules increase by millions, augmenting the mass and giving it the stimulus of heat which is only another name for motion.

Thus it appears that this point of vitalized matter, this microscopic moner, requires oxygen to build up and replace worn-out material, precisely the same as in the highest vertebrate animal.

The white corpuscles of our own blood are true moners, having amoeba-like motions and life. The red corpuscles of the blood are the oxygen carriers, and the white corpuscles have been seen to devour them.

The parallel is complete: From the highest to the lowest being, oxygen enters as a constituent, evolving substance, heat and motion. This primary substance is protoplasm, and without it life is impossible. It is the same restless matter, disobeying the laws of gravity in its movements and obeying only its own internal forces, whether examined in the moner or in the highest differentiated being. There are some biologists—and it must be confessed those who have given it long and laborious attention—who conclude that all the living matter in man is protoplasm, and composed chiefly of the white corpuscles.

These white corpuscles under the microscope may be seen to crawl across the glass like an amœba; and, as we have seen, they are most probably supplied with oxygen in the circulation by the red corpuscles.

In one way, then, or another, whatever the form of life, high or low, animal or vegetable, oxygen enters into the protoplasmic compound, giving rise to all the phenomena, either directly or indirectly, which we call life.

It is well that it bathes every square inch of our little planet, and it would be more in accordance with natural law, which has given us natural life, seeing it is "free as the air," if physicians would prescribe more of it.

